# Supplementary material for: Tailoring Cod Gelatin Structure and Physical Properties with Acid and Alkaline Extraction
Source: Polymers (Basel). 2019 Oct 21;11(10):1724. doi: 10.3390/polym11101724 (PMC6835541; doi:10.3390/polym11101724)
Supplement: Supplementary file 1 [file polymers-11-01724-s001.zip › polymers-621719-supplementary.pptx]

## Slide 1
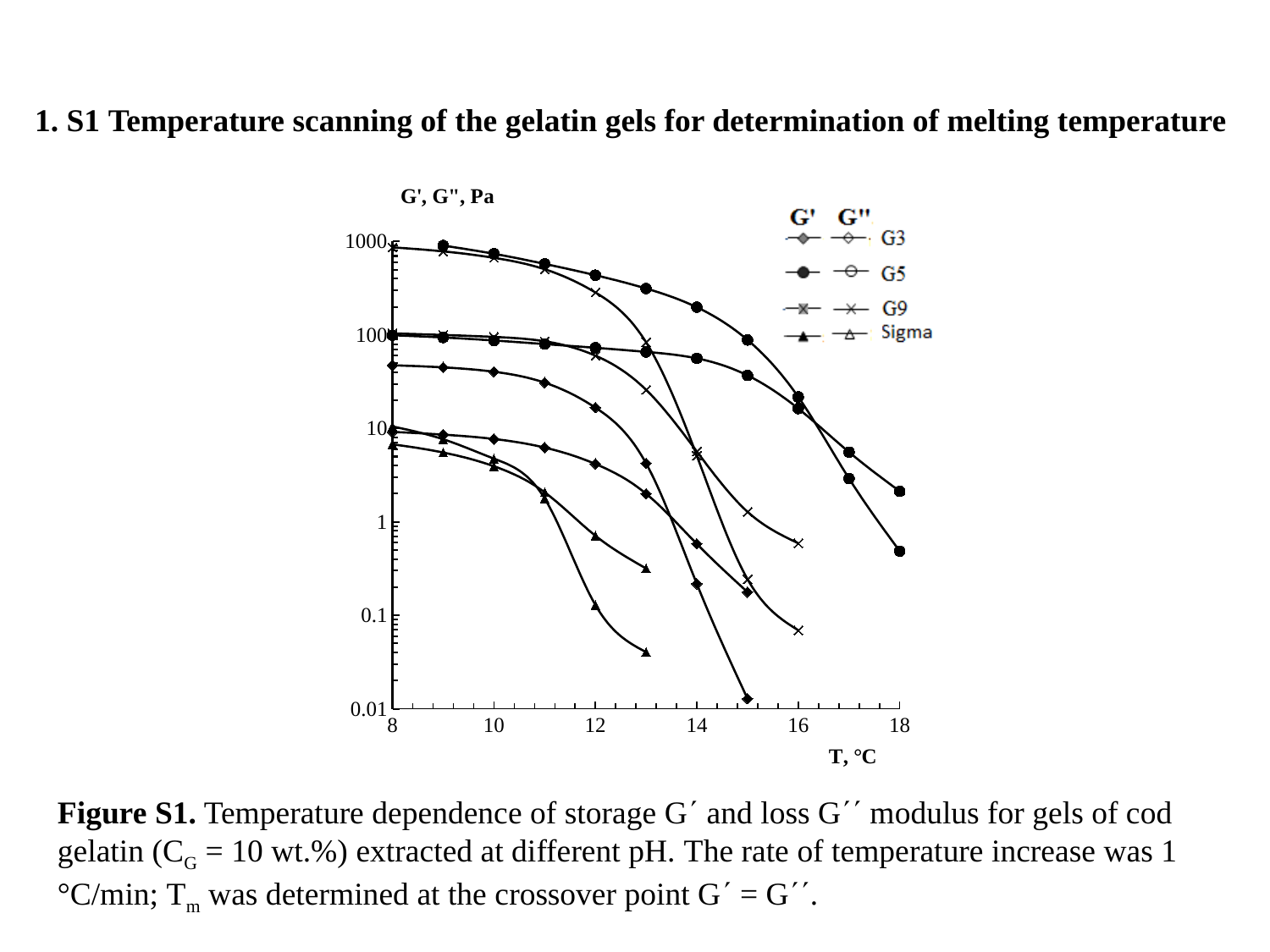

1. S1 Temperature scanning of the gelatin gels for determination of melting temperature
### Chart
| Category | рН 3 | рН 4 | рН 5 | рН 8 | рН 9 | Sigma | рН 3 | рН 4 | рН 5 | рН 8 | рН 9 | Sigma |
|---|---|---|---|---|---|---|---|---|---|---|---|---|Figure S1. Temperature dependence of storage G′ and loss G modulus for gels of cod gelatin (СG = 10 wt.%) extracted at different рН. The rate of temperature increase was 1 °C/min; Тm was determined at the crossover point G′ = G.

## Slide 2
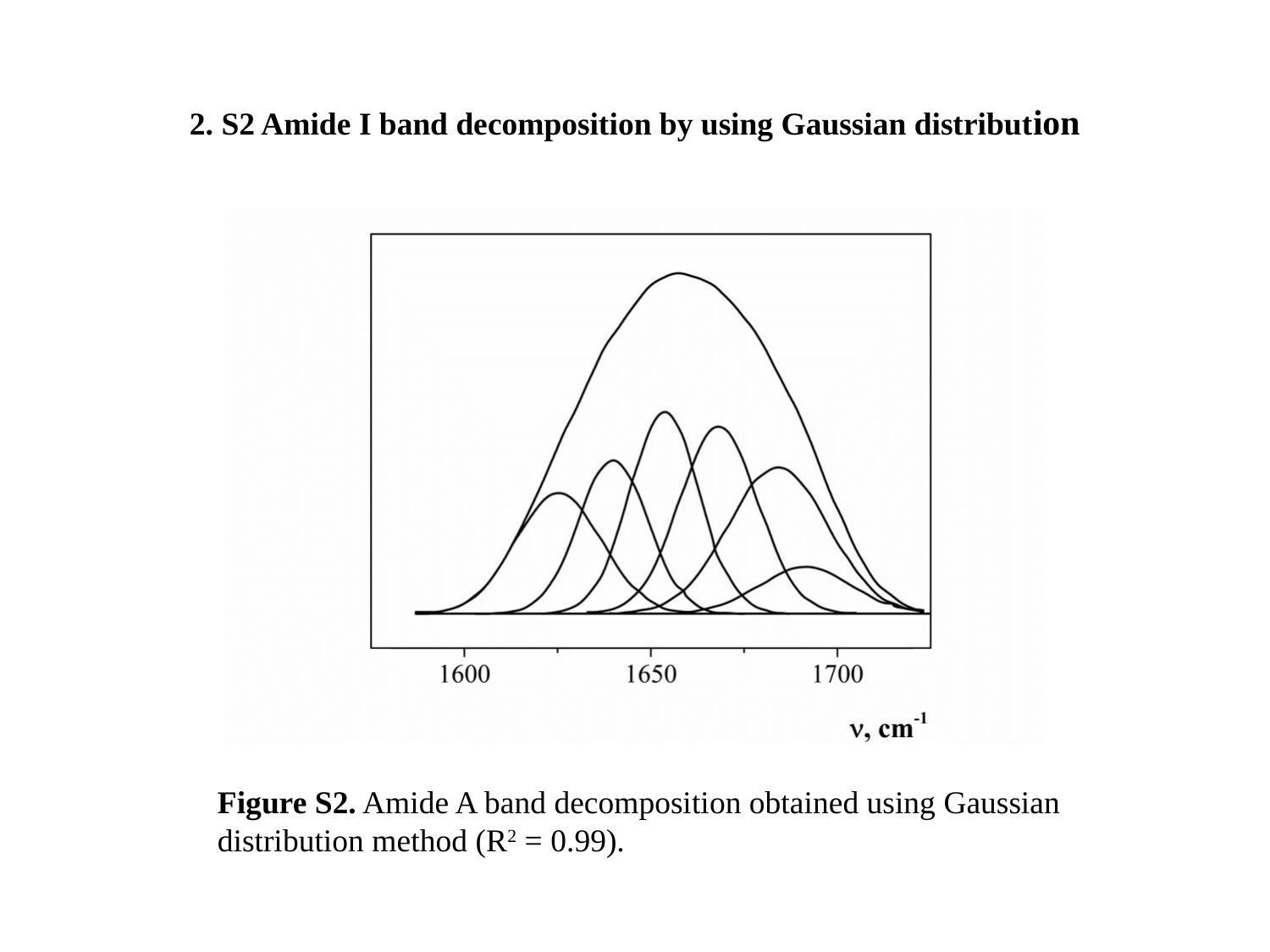

2. S2 Amide I band decomposition by using Gaussian distribution
Figure S2. Amide A band decomposition obtained using Gaussian distribution method (R2 = 0.99).
